# Analysis of oral cancer epidemiology in the US reveals state-specific trends: implications for oral cancer prevention

**DOI:** 10.1186/1471-2458-8-87

**Published:** 2008-03-10

**Authors:** Karl Kingsley, Susan O'Malley, Marcia Ditmyer, Michelle Chino

**Affiliations:** 1 Department of Biomedical Sciences, School of Dental Medicine, University of Nevada, Las Vegas, USA; 2 Department of Clinical Sciences and Professional Studies, School of Dental Medicine, University of Nevada, Las Vegas, USA; 3 Department of Environmental and Occupational Health, School of Public Health, University of Nevada, Las Vegas, USA

## Abstract

**Background:**

Downward trends have been observed in oral cancer incidence and mortality in the US over the past 30 years; however, these declines are not uniform within this population. Several studies have now demonstrated an increase in the incidence and mortality from oral cancers among certain demographic groups, which may have resulted from increased risks or risk behaviors. This study examines the underlying data that comprise these trends, to identify specific populations that may be at greater risk for morbidity and mortality from oral cancers.

**Methods:**

Oral cancer incidence and mortality data analyzed for this study were generated using the National Cancer Institute's Surveillance, Epidemiology and End Results (SEER) program.

**Results:**

While oral cancer incidence and mortality rates have been declining over the past thirty years, these declines have reversed in the past five years among some demographic groups, including black females and white males. Sorting of these data by state revealed that eight states exhibited increasing rates of oral cancer deaths, Nevada, North Carolina, Iowa, Ohio, Maine, Idaho, North Dakota, and Wyoming, in stark contrast to the national downward trend. Furthermore, a detailed analysis of data from these states revealed increasing rates of oral cancer among older white males, also contrary to the overall trends observed at the national level.

**Conclusion:**

These results signify that, despite the declining long-term trends in oral cancer incidence and mortality nationally, localized geographic areas exist where the incidence and mortality from oral cancers have been increasing. These areas represent sites where public health education and prevention efforts may be focused to target these specific populations in an effort to improve health outcomes and reduce disparities within these populations.

## Background

Although rates of oral cancer incidence and mortality in the US have declined over the past few decades, these declines have not been consistent or uniform within this population [1-4]. Collaborative reports using data from the National Cancer Institute (NCI), the Centers for Disease Control and Prevention (CDC) and the American Cancer Society (ACS) have found increases in the incidence of oral cancer among specific segments of the population, including minorities [5-7]. While many advances in treatment and diagnosis have been made over the past three decades, oral cancer remains the eighth leading cause of cancer death among US males [8] and the five-year survival rate has remained low and relatively unchanged [9,10]. Cancer remains the second leading cause of death in the US [11], and these observed increases in oral cancer provide compelling rationale for this study examining data underlying the general declining trends to elucidate which specific subsets of the population, as well as specific states or regions, that face increasing oral cancer rates.

Recently, studies of oral cancer epidemiology demonstrated statistically significant differences in oral cancer rates among population subgroups, including minorities and various age groups, and between genders [12]. One such study demonstrated that although incidence rates of oral cancer have been steadily decreasing among white males, incidence rates among older black males (>65 years old) have been increasing [13]. In addition, this study demonstrated that oral cancer rates among females, in particular, have increased [13]. Although these data provide some evidence of the disparities in oral cancer rates between these populations, a more detailed examination may identify states, metropolitan areas or communities, as well as additional population sub-groups within these areas, which are experiencing increases in oral cancer incidence or mortality.

This study will examine the underlying data that comprise the general trends, to identify specific populations within the US that may be at greater risk for morbidity and mortality from oral cancers. Epidemiology studies of oral cancer in Europe have found incidence and mortality rates have been declining steadily over the past few decades, similar to the trends found in the US, although more detailed analyses of the underlying data revealed that persistent upward trends were still present in a small subset of eastern European countries [14-18]. To perform a similar analysis for specific US states and counties, the NCI Surveillance, Epidemiology and End Results (SEER) website [19], a collaborative effort between the NCI and CDC, in conjunction with all US state registries, provides an interface for epidemiologists and other researchers to access and generate oral cancer statistics [20]. Due to the recently observed increases in oral cancer among particular segments of the US population, a more detailed analysis of the underlying data which comprise these general, long-term declining trends provides valuable information about significant short-term increases in specific geographic areas and among specific demographic groups.

## Methods

### Data sources

Population-based data for the US, specific to oral cancer, were obtained from the Surveillance, Epidemiology, and End Results (SEER) program. SEER provides cancer incidence and survival data from population-based cancer registries, representing approximately 25% of the US population [21]. All oral cancer statistics in this report are based on SEER incidence and National Center for Health Statistics (NCHS) mortality statistics, which consisted of cancers of the oral cavity and pharynx, including the lip, oral cavity and pharynx [22].

### Incidence

Oral cancer incidence rates for each year between 1975 and 2004 were obtained from SEER, age-adjusted to the year 2000 standard US population. The overall incidence trends for each time period (1975–2004; 1995–2004; 2000–2004) were then calculated and subsequently graphed based on these data, dividing the most recent incidence rate by each specific earlier rate.

### Mortality

Oral cancer mortality rates for each year between 1975 and 2004, age-adjusted to the year 2000 standard US population, were also obtained from SEER. Data qualified for inclusion in SEER as oral cavity and pharyngeal cancer if the underlying cause of death was specific for oral cancers [20]. The overall mortality trends over time for each time period (1975–2004; 1995–2004; 2000–2004) were calculated and graphed based on data from 1975–2004, dividing the most recent mortality rate by earlier rates.

### Annual percent change (APC) 1999–2003

Recent trend data in death rates from oral cancer from individual US states were calculated from the State Cancer Registries in SEER using the Joinpoint Regression Progression and are expressed as the APC over the reported trend period (1999–2003). Current annual death rates of oral cancers from individual US states were similarly obtained and the most recent data available (2003, 2004) at the time of article preparation were reported. Data were exported to Microsoft Excel, sorted in ascending order and graphed.

### US state data

Historical mortality data for cancers of the oral cavity and pharynx from selected US states, including Nevada, Idaho, North Dakota, and North Carolina, were calculated by NCI SEER*Stat, from data provided by the National Vital Statistics System public use data file. Trends are based upon analysis calculated using the Joinpoint Regression Program statistical software program, which models the natural logarithm of the rates, identifying years at which any given trend changes, connecting these years graphically by a series of straight line segments [23,24].

## Results

### Age-adjusted incidence and mortality rates

**Figure 1:**
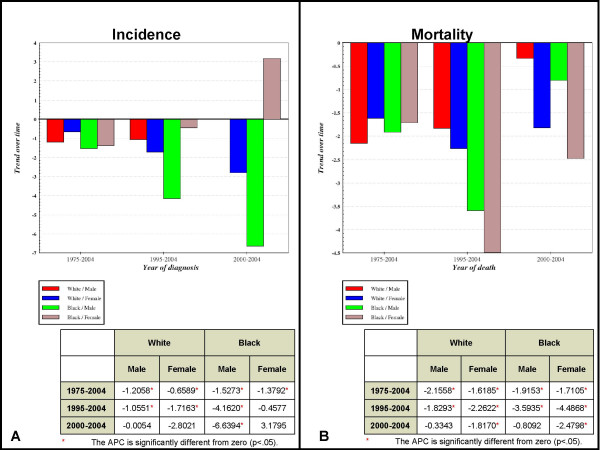
**Incidence and mortality trends for oral cancer in the US**. Trends for the incidence (A) and mortality (B) rates of oral cancer cases reported to the SEER program from 1975–2004 in the US were sorted by time period (30 year, 10 year and 5 year), and by race/ethnicity and gender.

Age-Adjusted Incidence Rates (AAIR) and Age-Adjusted Mortality Rates (AAMR) were generated and sorted by race and gender to gather more detailed information regarding oral cancer trends (Fig. 1). The oral cancer incidence and mortality trends were then further delineated into three, distinct time periods, 1975–2004 (30 year), 1995–2004 (10 year) and 2000–2004 (5 year), to allow for greater specificity within the overall temporal trend analysis.

#### Age-adjusted incidence rates (AAIR)

Analysis of the AAIR data revealed an overall declining trend in oral cancer incidence over the past 30 years (Fig. 1A). More specifically, over the past 30 years, oral cancer incidence has declined among white males (-1.21%), white females (-0.66%), black males (-1.53%) and black females (-1.38%), although these observed declines have not been uniform across time or demographic groups. For example, although the incidence of oral cancer among black males has declined over the past 30 years, the temporal stratification of these data revealed that this decline was greatest over the past five years (-6.64%). Furthermore, this stratification also revealed a contrasting trend; the incidence of oral cancer among black females rose from -1.38%, over the entire 30 year period, to +3.18% during the most recent five year period.

#### Age-adjusted mortality rates (AAMR)

Analysis of the AAMR data also revealed an overall declining trend in oral cancer mortality over the past 30 years (Fig. 1B). Although overall mortality decreased over 30 years for all groups analyzed, white males (-2.16%), white females (-1.62%), black males (-1.92%) and black females (-1.71%), more specific temporal analysis of oral cancer mortality revealed at least two distinct trends. First, the decreases in mortality were greatest over the last 10 year period compared to the last 30 years and much less pronounced over the more recent five year period. This trend was observed for white females, black males, and black females, but not white males. The second trend, found only among white males, revealed that mortality, although still declining, was declining by ever smaller amounts over each time period: 30 years (-2.16%), 10 years (-1.83%), and five years (-0.33%).

### Geographic distribution

**Figure 2:**
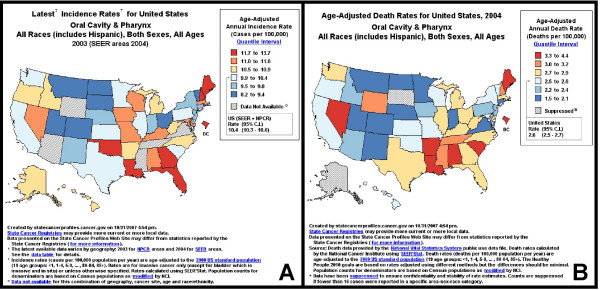
**Geographic distribution of current incidence and mortality rates for oral cancer in the US**. Age-adjusted incidence (A) and mortality (B) rates for all US states, for all races, all ages and both sexes, were ranked in quantiles, based upon cases per 100,000 and mapped: created by statecancerprofiles.cancer.gov.

To determine if the temporal shifts observed in oral cancer incidence and mortality were associated with specific geographic regions or states, AAIR and AAMR were generated for all US states (Fig. 2). AAIR and AAMR data were then further delineated into quantile intervals to highlight the rates for each state relative to the US averages, from highest (red) to lowest (dark blue).

#### AAIR geographic distribution

Analysis of the AAIR geographic distribution data identified seven states in the highest quantile (11.7 to 13.7 cases per 100,000), the District of Columbia (13.7), Oklahoma (12.7), Louisiana (12.7), Georgia (12.6), Florida (12.5), Maine (12.2) and New Hampshire (11.8) (Fig. 2A). Seven additional states were identified within the second highest quantile (11.0 to 11.6), Maryland (11.6), Alabama (11.5), Wisconsin (11.5), Missouri (11.4), Kentucky (11.4), Nevada (11.2) and Massachusetts (11.0). All states within the two highest quantiles were located in the Eastern and Central zones, with the exception of Nevada (Pacific).

#### AAMR geographic distribution

Analysis of the AAMR geographic distribution data revealed seven states within the highest quantile (3.3 to 4.4 deaths per 100,000), the District of Columbia (4.4), Arkansas (3.5), South Carolina (3.5), Louisiana (3.4), Alabama (3.3), Maine (3.3) and Nevada (3.3) (Fig. 2B). The second highest quantile was comprised of four states, New Hampshire (3.2), Wyoming (3.2), Mississippi (3.0) and Tennessee (3.0). Once again, the majority of states within the two highest quantiles were located in the Eastern and Central zones of the US, with the exception of Nevada (Pacific) and Wyoming (Mountain).

### Annual percent change in US states

The graphic organization of specific states with the highest levels of oral cancer incidence and mortality provides significant information regarding the geographic regions which are associated with these highest levels. This information does not, however, delineate the areas which have high levels of oral cancer incidence and mortality that are slowly decreasing over time and those that are increasing. To make this determination, the most recent five year interval was selected to provide a more detailed temporal and geographic breakdown of the states reporting oral cancer incidence or mortality within the two highest quantiles, to determine if the annual percent change (APC) was decreasing at a slower rate, or increasing over time (Table 1).

**Table 1:** Comparison of annual percent change (APC) in oral cancer mortality among US states with higher than average incidence and mortality US states from the highest two quantiles of current oral cancer incidence and mortality were sorted in descending order to compare with recent (1999–2003) APC rate trends in mortality. Among these, three states were found to have increasing APC, Maine, Nevada, and Wyoming.

**US states (elevated incidence)**	**APC trend (recent)**	**Mortality rate (current)**	**Incidence rate (current)**
District of Columbia	-4.6%	4.4/100,000	13.7/100,000
Oklahoma	-1.5	2.5	12.7
Louisiana	-1.2	3.4	12.7
Georgia	-2.8	2.9	12.6
Florida	-3.1	2.9	12.5
**Maine**	**+2.2**	3.3	12.2
New Hampshire	-0.9	3.2	11.8
Maryland	-2.9	2.5	11.6
Alabama	-1.2	3.3	11.5
Wisconsin	-1.5	2.6	11.5
Kentucky	-1.5	2.9	11.4
**Nevada**	**+4.6**	3.3	11.2
Massachusetts	-1.0	2.8	11.0
United States	-1.1%	2.6/100,000	10.4/100,000
**US states (elevated mortality)**	APC trend (recent)	Mortality rate (current)	Incidence rate (current)
District of Columbia	-4.6%	4.4/100,000	13.7/100,000
Arkansas	0.0	3.5	10.6
South Carolina	-3.7	3.5	10.1
Louisiana	-1.2	3.4	12.7
Alabama	-1.2	3.3	11.5
**Maine**	**+2.2**	3.3	12.2
**Nevada**	**+4.6**	3.3	11.2
New Hampshire	-0.9	3.2	11.8
**Wyoming**	**+0.1**	3.2	N/Q*
Mississippi	-0.1	3.0	N/Q*
Tennessee	-1.1	3.0	N/Q*
United States	-1.1%	2.6/100,000	10.4/100,000

*N/Q Data not provided because it did not meet United States Cancer Statistics (USCS) data quality standards for one or more years during the rate period of data collection

#### APC in US states with elevated incidence

Analysis of APC from the stratified AAIR data identified 14 states that were in the highest quantiles for oral cancer incidence (Table 1). Of these states, 12 were found to have negative APC, which indicates a continuing decreasing trend in oral cancer incidence over the most recent five-year interval, although most of these decreases were comparatively lower than observed over the longest time interval (30 years). Two states which did not follow this trend, however, and that were found to have increasing APC, were Maine (+2.2%) and Nevada (+4.6%).

#### APC in US states with elevated mortality

Analysis of APC from the stratified AAMR data identified 11 states that were in the highest quantiles for oral cancer mortality (Table 1). Of these states, eight were found to have negative APC, which suggests a continuing decreasing trend in oral cancer mortality, although most of the decreases were also comparatively lower than observed over the longest time interval (30 years). Three states, however, were found to have increasing APC, Maine (+2.2%), Nevada (+4.6%) and Wyoming (+0.1%).

#### US states with positive APC

**Figure 3:**
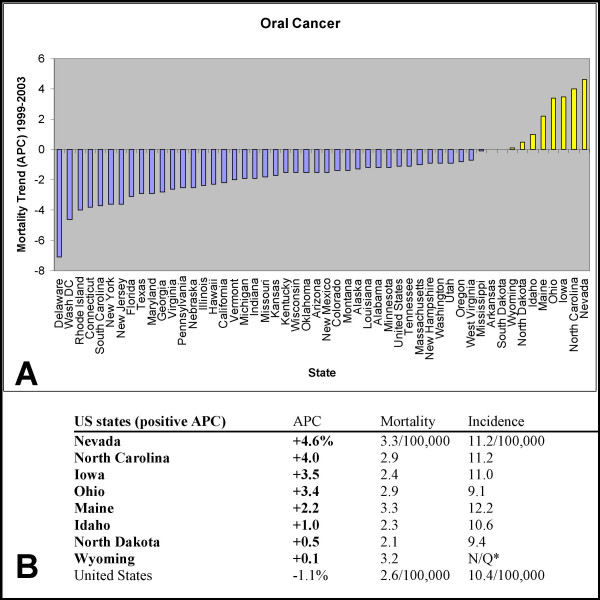
**Annual percent change (APC) in mortality rates for oral cancer, 1999–2003**. Annual Percent Change (APC) for the age-adjusted mortality rates for cancer of the oral cavity and pharynx, for all ages, genders and races, sorted by US state, 1999–2003, were created by statecancerprofiles.cancer.gov, using NCI SEER*Stat and sorted. Eight states were identified with positive, increasing APC using this method. *N/Q: Data not provided because it did not meet United States Cancer Statistics (USCS) data quality standards for one or more years during the rate period of data collection.

Although we identified states with positive APC, in the highest quantiles for both oral cancer incidence and mortality (Maine, Nevada), one additional state was found to have positive APC, which was only found among the states with elevated mortality. To determine if other states had increasing APC, but were not among the states with the highest overall levels of oral cancer incidence or mortality, we expanded this analysis to include data for all US states. This analysis revealed that eight states had increasing APC in oral cancer mortality (Fig. 3). These states were Nevada (+4.6%), North Carolina (+4.0%), Iowa (+3.5%), Ohio (+3.4%), Maine (+2.2%), Idaho (+1.0%), North Dakota (+0.5%) and Wyoming (+0.15), only three of which were among the states with either the highest oral cancer incidence or mortality.

**Figure 4:**
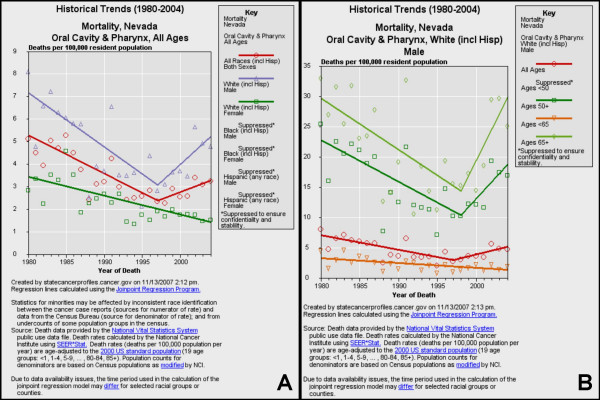
**Historical trends for oral cancer mortality in Nevada**. Historical trends (1980–2004) of mortality from oral cancer were sorted by race/ethnicity and gender (A) using NCI SEER*Stat and regression lines calculated using the Joinpoint Regression Program. Increasing trend in mortality among white males (1997–2004) was further delineated by age (B).

**Figure 5:**
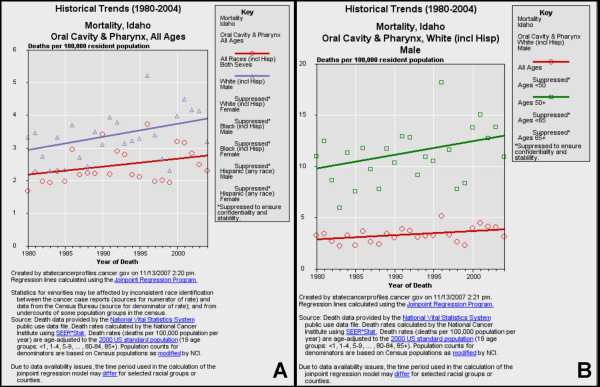
**Historical trends for oral cancer mortality in Idaho**. Historical trends (1980–2004) of mortality from oral cancer were sorted by race/ethnicity and gender (A) using NCI SEER*Stat and regression lines calculated using the Joinpoint Regression Program. Mortality among white males was sorted further by age (B).

**Figure 6:**
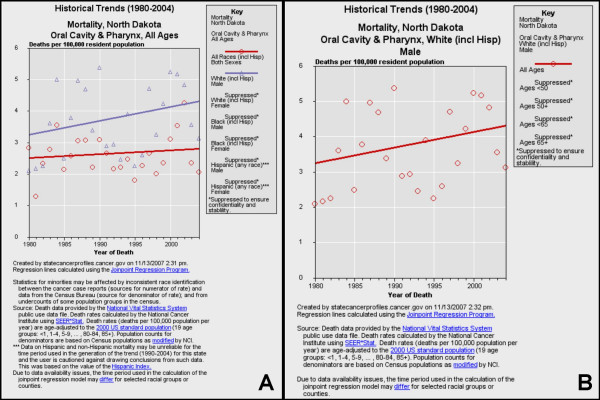
**Historical trends for oral cancer mortality in North Dakota**. Historical trends (1980–2004) of mortality from oral cancer were sorted by race/ethnicity and gender (A) using NCI SEER*Stat and regression lines calculated using the Joinpoint Regression Program. Mortality among white males was then stratified by age (B).

**Figure 7:**
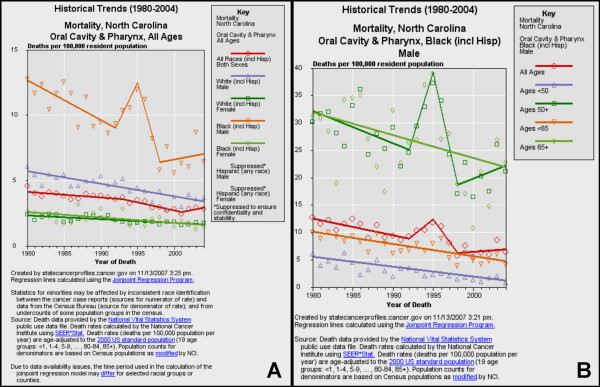
**Historical trends for oral cancer mortality in North Carolina**. Historical trends (1980–2004) of mortality from oral cancer were sorted by race/ethnicity and gender (A) using NCI SEER*Stat and regression lines calculated using the Joinpoint Regression Program. The increasing trend among black males was sorted further by age (B).

Having identified eight states with recent increasing or positive trends (APC), a more detailed analysis of each of these states was performed to further examine these trends within each state. The more detailed analysis of each state, year-to-year, spanning a 25 year period revealed significant, increasing trends in only four of these states, Nevada (Fig. 4), Idaho (Fig. 5), North Dakota (Fig. 6) and North Carolina (Fig. 7).

#### Nevada

Oral cancer mortality data for Nevada, the state with the highest five-year APC (+4.6%), were stratified by race and gender (Fig. 4). The analysis revealed that although the rates of oral cancer were decreasing for many years (1980–1997), a distinct upward trend was noted (1998–2004). Moreover, this trend was most closely associated with white males (Fig. 4A). The data for white males were then further stratified by age, revealing that the recent upward trends in oral cancer mortality were almost exclusively associated with white males over 50, and especially with white males over 65 (Fig. 4B).

#### Idaho

Oral cancer mortality data for Idaho, another state identified with a positive five-year APC (+1.0), were also sorted by both race and gender (Fig. 5). The results from this stratification revealed that the rates of oral cancer in Idaho have been slowly increasing for many years (1980–2004). More specifically, this trend was associated almost exclusively with white males (Fig. 5A), with too few data points to support trend analysis for any other race or gender grouping. Separating the data for white males by age revealed that this upward trend was associated with white males over 50, but not older than 65 (Fig. 5B).

#### North Dakota

Oral cancer mortality data for North Dakota, a state with a small, positive five-year APC (+0.5%), were also sorted by race and gender (Fig. 6). The results from this analysis revealed that rates of oral cancer in North Dakota have been slowly increasing over many years (1980–2004) and the increases were also associated with white males (Fig. 6A). After separating these data by age, the results did not find this trend was associated with any specific age group (Fig. 6B).

#### North Carolina

Oral cancer mortality data for North Carolina, a state with a positive five-year APC (+4.5%), were also sorted by race and gender (Fig. 7). The results from this analysis, however, revealed that the recent increasing trend in oral cancer mortality (2001–2004) was primarily associated with black males (Fig. 7A). After the data for black males were further stratified by age, the upward trend was most closely associated with black males over 50, but not older than 65 (Fig. 7B).

## Discussion

Although cancer ranks as the second leading cause of death in the United States, after heart disease, and remains an important problem facing public health professionals, the overall rates of cancer deaths have been steadily declining over the past few decades [25]. While this declining trend is welcome news for the general population and health professionals, it does not accurately describe the details which underlie these trends in which rates for some types of cancer have decreased significantly, while rates of other cancers have displayed opposing, increasing trends [11]. For example, although rates of lung cancer have steadily declined for decades, cancers of the liver and thyroid have increased over the same period [3,11,26]. In the same fashion, the overall declining rates observed for oral cancer may obfuscate the underlying data which suggest that while the rates are declining among whites, they may be simultaneously increasing among other ethnic or demographic groups, such as blacks and females [13,27].

To accurately understand the changes in oral cancer incidence and mortality, it is important to examine not only the composite data which describe the general trends for the US population over many years, but also to scrutinize the primary core data which convey more detailed information. For example, these core data may include shorter intervals and year-by-year trends, as well as demographic and geographic breakdowns. Although previous reports have noted that oral cancer incidence and mortality rates are not uniform across demographic groups [13,27], this report is among the first to describe that oral cancer rates may be increasing over the short term, and that these increases are restricted to a small subset of states and particular demographic groups.

Previous studies have described an overall declining trend in oral cancer incidence and mortality with the understanding that these decreases were found primarily among whites, and were not offset by smaller increases among other demographic groups [20,28]. This report, however, provides evidence of three distinct trends, not previously articulated. First, although oral cancer incidence and mortality have declined over the past thirty years, with the most significant declines observed over the past ten years, a reversal of these trends has emerged from the short-term (over the past five years) trend analysis, which may signify an important development in the epidemiology of this cancer. Next, this report provides a geographic profile of oral cancer rates over time, revealing that although oral cancer rates are continuing to decline in most states, they are now in fact increasing in a small subset of states. Finally, in-depth stratification of data from these specific states revealed that oral cancer rates are increasing almost exclusively among older white males in three of these states, in sharp contrast to the general national trends.

The identification of differential oral cancer trends among specific geographic areas and demographic groups in the US could indicate a shift in the epidemiology of this cancer. A recent large-scale study among European countries revealed similar temporal and geographic trends [18]. For example, although oral cancer incidence and mortality has steadily declined in Europe as a whole since the 1980s, more detailed analysis by geographic region (country) revealed that mortality was rising in a subset of eastern European countries, most notably in Bulgaria, Romania, Hungary, Slovakia and Slovenia [16,17]. Based upon these observations, the study authors speculated that the temporal and geographic nature of these patterns was related to changes in exposure to the two major risk factors for oral cancer, alcohol and tobacco. These items became more readily available and widely disseminated in these areas following the disintegration and break-up of the Soviet Union [18].

Perhaps the increasing oral cancer trends identified in this study, in specific states and among specific demographic groups, are related to identifiable trends in oral cancer risk factors and behaviors, such as increased tobacco use or alcohol consumption, as was found in eastern European countries. The most recent Behavioral Risk Factor Surveillance System (BRFSS) data confirms that six of the eight states identified in this report with increasing trends in oral cancer mortality were also among the states with higher than average rates of current smokers, which include Ohio, Nevada, North Carolina, Wyoming, Iowa and Maine [29]. Moreover, these states were also among the states with higher than average rates of heavy alcohol consumers, with the exception of North Carolina. Although these data suggest a correlation between alcohol and tobacco consumption patterns and oral cancer in these areas and among these demographic groups, the BRFSS data also provide some conflicting evidence, revealing that the states with the highest levels of current smokers and heavy alcohol consumers (Kentucky and Wisconsin, respectively) were not among those states with increasing rates of oral cancer incidence and mortality, but rather have decreasing rates, indicating that other risk factors may also be significant contributing factors.

Although tobacco and alcohol consumption are the main risk factors for developing oral cancer, implicated in as many as 90 to 95% of head and neck cancers, other potential risk factors have recently emerged [30]. For instance, evidence for the role of infectious agents in the etiology of oral cancers has been mounting, demonstrating that oral infection with high-risk human papillomavirus (HPV) may not only increase the risk of developing oral cancer, but may also contribute to its progression [31,32]. Other infectious agents and immune modulators, such as infection with the human immunodeficiency virus (HIV) and immune suppression, induced mainly via pharmacologic means to prevent rejection of transplanted organs, also significantly increase the risk of developing oral cancer [12]. In addition, recent evidence demonstrates that nutrition may play an important role in retarding the development and progression of oral cancers, revealing a nearly 50% reduction in oral cancer risk for each additional portion of fruits or vegetables consumed per day, even among tobacco and alcohol consumers [33-36]. Identifying those demographic groups and geographic areas experiencing increases in oral cancer will help direct public health research to understand how and why these rates may be increasing.

## Conclusion

It is imperative that further analysis of the contributing factors that underlie these temporal and geographic trends be undertaken. This information may be indispensable to public health professionals as they strive to design population-specific prevention and education programs, which are often funded and implemented at the local, regional and state levels. Because many of the lifestyle behaviors which contribute to oral cancer risk are possible to impact through public health education and prevention strategies, more effective targeting of public health monies and efforts, towards the specific geographic regions and demographic populations which face these increased risks, may help to reverse these disturbing trends of increasing oral cancer, as outlined in this study.

## Competing interests

The author(s) declare they have no competing interests.

## Authors' contributions

KK performed the statistical analyses. SO and MD assisted with the interpretation and analysis of data generated and made significant contributions to the writing and editing of this manuscript. MC and KK conceived and coordinated the design of this project. All authors have read and approved the final version of this manuscript.

## Pre-publication history

The pre-publication history for this paper can be accessed here:



## References

[B1] Wingo PA, Ries LA, Rosenberg HM, Miller DS, Edwards BK (1998). Cancer incidence and mortality, 1973–1995: a report card for the US. Cancer.

[B2] Wingo PA, Ries LA, Giovino GA, Miller DS, Rosenberg HM, Shopland DR, Thun MJ, Edwards BK (1996). Annual report to the nation on the status of cancer, 1973-with a special section on lung cancer and tobacco smoking. J Natl Cancer Inst.

[B3] Ries LA, Wingo PA, Miller DS, Howe HL, Weir HK, Rosenberg HM, Vernon SW, Cronin K, Edwards BK (2000). The annual report to the nation on the status of cancer, 1973–1997 with a Special Section on Colorectal Cancer. Cancer.

[B4] Edwards BK, Howe HL, Ries LA, Thun MJ, Rosenberg HM, Yanick R, Wingo PA, Jemal A, Feigal EG (1999). Annual report to the nation on the status of cancer, 1973-featuring implications of age and aging on U.S. cancer burden. Cancer.

[B5] Levi F, Lucchini F, Negri E, Boyle P, La Vecchia C (2001). Changed trends of cancer mortality in the elderly. Ann Oncol.

[B6] Ries LAG, Eisner MP, Kosary CL, Hankey BF, Miller BA, Clegg L, Mariotto A, Feuer EJ, Edwards BK, eds (2005). SEER Cancer Statistics Review, 1975–2002. National Cancer Institute.

[B7] Morse DE, Kerr AR (2006). Disparities in oral and pharyngeal cancer incidence, mortality and survival among black and white Americans [Erratum in: J Am Dent Assoc 137:447]. J Am Dent Assoc.

[B8] Jemal A, Siegel R, Ward E, Murray T, Xu J, Smigal C, Thun MJ (2006). Cancer statistics, 2006. CA Cancer J Clin.

[B9] Ries LAG, Eisner MP, Kosary CL, Hankey BF, Miller BA, Clegg L, Edwards BK, eds (2001). SEER Cancer Statistics Review, 1973–1998. National Cancer Institute.

[B10] Silverman S (2001). Demographics and occurrence of oral and pharyngeal cancers. The outcomes, the trends, the challenge. J Am Dent Assoc.

[B11] Jemal A, Siegel R, Ward E, Murray T, Xu J, Thun MJ (2007). Cancer statistics, 2007. CA Cancer J Clin.

[B12] Swango PA (1996). Cancers of the oral cavity and pharynx in the United States: an epidemiologic overview. J Public Health Dent.

[B13] Shiboski CH, Shiboski SC, Silverman S (2000). Trends in oral cancer rates in the United States, 1973–1996. Community Dent Oral Epidemiol.

[B14] Bray I, Brennan P, Boffetta P (2000). Projections of alcohol- and tobacco-related cancer mortality in Central Europe. Int J Cancer.

[B15] Levi F, Lucchini F, Negri E, Boyle P, La Vecchia C (2003). Mortality from major cancer sites in the European Union, 1955–1998. Ann Oncol.

[B16] La Vecchia C, Levi F, Franceschi S (2000). Epidemiology of cancer with a focus on Europe. J Epidemiol Biostat.

[B17] La Vecchia C, Franceschi S, Levi F (2003). Epidemiological research on cancer with a focus on Europe. Eur J Cancer Prev.

[B18] La Vecchia C, Lucchini F, Negri E, Levi F (2004). Trends in oral cancer mortality in Europe. Oral Oncol.

[B19] National Cancer Institute, Surveillance, Epidemiology and End Results Cancer Query Systems. http://seer.cancer.gov/canques/.

[B20] Davies L, Welch HG (2006). Epidemiology of head and neck cancer in the United States. Otolaryngol Head Neck Surg.

[B21] National Cancer Institute, Surveillance, Epidemiology and End Results Database (NCI-SEER). http://seer.cancer.gov/about/.

[B22] Ries LAG, Melbert D, Krapcho M, Mariotto A, Miller BA, Feuer EJ, Clegg L, Horner MJ, Howlader N, Eisner MP, Reichman M, Edwards BK, eds (2007). SEER Cancer Statistics Review, 1975–2004. National Cancer Institute.

[B23] Kim HJ, Fay MP, Feuer EJ, Midthune DN (2001). Permutation tests for joinpoint regression with applications to cancer rates [Erratum in: Stat Med 20:655]. Stat Med.

[B24] National Cancer Institute Joinpoint Regression Program (version 3.0). http://seer.cancer.gov/seerstat.

[B25] Mokdad AH, Marks JS, Stroup DF, Gerberding JL (2005). Actual causes of death in the United States, 2000 [Erratum in: JAMA 293:293–294]. JAMA.

[B26] Hodgson NC, Button J, Solorzano CC (2004). Thyroid cancer: is the incidence still increasing?. Ann Surg Oncol.

[B27] Shavers VL, Harlan LC, Winn D, Davies WW (2003). Racial/ethnic patterns of care for cancers of the oral cavity, pharynx, larynx, sinuses, and salivary glands. Cancer Metastasis Rev.

[B28] Weir HK, Thun MJ, Hankey BF, Ries LA, Howe HL, Wingo PA, Jemal A, Ward E, Anderson RN, Edwards BK (2000). Annual report to the nation on the status of cancer, 1975-featuring the uses of surveillance data for cancer prevention and control [Erratum in: J Natl Cancer Inst 2003, 95:1641]. J Natl Cancer Inst.

[B29] Kuiper N, Malarcher A, Bombard J, Maurice E, Jackson K (2005). State-specific prevalence of cigarette smoking and quitting among adults- United States, 2004. MMWR.

[B30] Blot WJ, McLaughlin JK, Winn DM, Austin DF, Greenberg RS, Preston-Martin S, Bernstein L, Schoenberg JB, Stemhagen A, Fraumeni JF (1988). Smoking and drinking in relation to oral and pharyngeal cancer. Cancer Res.

[B31] Kingsley K, Johnson D, O'Malley S (2006). Transfection of oral squamous cell carcinoma with human papillomavirus-16 induces proliferative and morphologic changes independent of cellular adhesion *in vitro*. Cancer Cell Int.

[B32] Reddout N, Christensen T, Bunnell A, Jensen D, Johnson D, O'Malley S, Kingsley K (2007). High risk HPV types 18 and 16 are potent modulators of oral squamous cell carcinoma phenotypes *in vitro*. Infect Agent Cancer.

[B33] Le Marchand L (2002). Cancer prevention effects of flavonoids – a review. Biomed Pharmacother.

[B34] Hsu S, Singh B, Schuster G (2004). Induction of apoptosis in oral cancer cells: agents and mechanisms for potential therapy and prevention. Oral Oncol.

[B35] Pavia M, Pileggi C, Nobile CGA, Angelillo IF (2006). Association between fruit and vegetable consumption and oral cancer: a meta-analysis of observational studies. Am J Clin Nutr.

[B36] King M, Chatelain K, Farris D, Jensen D, Pickup J, Swapp A, O'Malley S, Kingsley K (2007). Oral squamous cell carcinoma proliferative phenotype is modulated by proanthocyanidins: a potential prevention and treatment alternative for oral cancer. BMC Complement Altern Med.

